# Sample environment for simultaneous quasi-elastic neutron scattering and Raman spectroscopy experiments demonstrated on polymer films under changing humidity and temperature

**DOI:** 10.1107/S1600576725008519

**Published:** 2025-11-04

**Authors:** Lucas P. Kreuzer, Marcell Wolf, Friederike Ganster, Christopher J. Garvey, Adrian Stephan, Marie Betker, Fanni Juranyi, Peter Müller-Buschbaum

**Affiliations:** a Heinz Maier-Leibnitz-Zentrum, Technical University of Munich, Lichtenbergstraße 1, 85748 Garching, Germany; b Technical University of Munich, TUM School of Natural Sciences, Department of Physics, Chair for Functional Materials, James-Franck-Str. 1, 85748 Garching, Germany; c Deutsches Elektronen Synchrotron DESY Notkestraße 85 22607 Hamburg Germany; d PSI Center for Neutron and Muon Sciences, 5232 Villigen PSI, Switzerland; Universität Duisburg-Essen, Germany

**Keywords:** quasi-elastic neutron scattering, Raman spectroscopy, polymer films, *in situ* hydration, sample environments

## Abstract

Understanding the dynamic and structural properties of matter across different timescales and length scales is essential for elucidating their functionality. We focus here on soft-matter systems, which are typically particularly sensitive to relative humidity (RH) and temperature, necessitating well controlled experimental conditions. Quasi-elastic neutron scattering (QENS) and Raman spectroscopy offer complementary insights: QENS accesses diffusional dynamics, which are stochastic motions, while Raman spectroscopy provides information on periodic vibrational dynamics. To enable simultaneous QENS and Raman measurements on *e.g.* polymer films under controlled and variable RH and temperature, we have developed a dedicated sample environment (SE), which in the presented experiments covers an RH range of 5–85% and a temperature range of 20–50°C. This SE features a 3D-printed spherical chamber including a sample holder for films, a Raman spectrometer, a custom-built gas-flow system and a thermostat. The chamber can accommodate large samples (up to 5 × 5 cm) and provides space for additional measurement equipment. The 3D-printing technique allows the integration of channels into the chamber walls, which are used for cooling and heating, resulting in highly homogeneous temperature distributions. This is key to achieving a uniform humidity in the chamber. The chamber works with a variety of solvents and their mixtures, like heavy water or ethanol. We demonstrate the feasibility of this setup through simultaneous QENS and Raman measurements on poly(3,4-ethylene di­oxy­thio­phene):poly(styrene­sulfonate) films on Si substrates, confirming that the chamber provides a stable and low scattering background environment.

## Abstract 2

We present a sample environment for simultaneous quasi-elastic neutron scattering and Raman spectroscopy experiments demonstrated on soft-matter films under variable and controlled relative humidity and temperature.

## 1. Introduction

Correlating and understanding the dynamic and structural properties of hydrated soft-matter systems spanning over large timescales and length scales [picoseconds to nano­seconds and ångströms to nanometres accessible with quasi-elastic neutron scattering (QENS)] is crucial to resolving their functionality. In the literature numerous examples can be found: The dynamic structure of the hydration shell strongly influences the conformation and function of macromolecules (Bellissent-Funel *et al.*, 2016 ▸; Laage *et al.*, 2017 ▸). In responsive systems, the dynamics of hydration water are key for the response of a polymer towards external stimuli, *e.g.* temperature or pressure (Aseyev *et al.*, 2011 ▸; Kojima, 2018 ▸; Niebuur *et al.*, 2019 ▸). The transport properties of membranes or hydrogels are linked to the dynamic properties of the polymers and hydration water, and generally the interaction with water, especially with hydration water, is of fundamental importance as it greatly influences the polymer’s mobility and its nanostructure and determines the ratio of exposed hydrophilic and hydrophobic functional groups (Kojima, 2018 ▸; Solhi *et al.*, 2023 ▸; Etale *et al.*, 2023 ▸; Sjöstrand *et al.*, 2024 ▸; Kreuzer *et al.*, 2025 ▸).

QENS is a powerful technique to study the diffusional properties of soft-matter systems, typically probing trans­lational and rotational motions of hydrogen-bearing species. The neutron wavelength and energy are in an ideal range to probe dynamics occurring on a pico- to nanosecond scale, thereby covering relevant timescales for water and polymer dynamics. The scattered intensity is measured as a function of momentum transfer *Q* and energy transfer *E*, thereby providing valuable spatiotemporal information. This means that QENS not only captures the timescales of molecular motion but also reveals the characteristic length scales at which these dynamics occur. Furthermore, the large neutron scattering cross section of hydrogen makes QENS especially sensitive to hydrogenous systems and allows the direct probing of diffusive motions of water and polymers (Wood *et al.*, 2007 ▸; Frölich *et al.*, 2009 ▸; Colmenero & Arbe, 2013 ▸; Richter & Kruteva, 2019 ▸; Kruteva, 2021 ▸; Guccini *et al.*, 2022 ▸; Kreuzer *et al.*, 2025 ▸).

In contrast, Raman spectroscopy measures vibrational dynamics, thus providing access to the structural and chemical environment of the probed system. The accessible timescales depend on the Raman shift that can be probed and are typically in the range of femtoseconds (*e.g.* a Raman spectrum measured within a range of 500 to 4000 cm^−1^ corresponds to timescales of 67 to 8 fs). Note that the probed energy ranges for a cold neutron spectrometer and Raman spectroscopy can overlap (on the neutron energy gain side if the sample temperature is high enough). Close to room temperature, reasonable intensity can be obtained up to approximately 100–150 meV, but the energy resolution is not sufficient to detect the occurrence of peak shifts. Thus the focus is rather on the quasi-elastic regime, where diffusive dynamics appear (Niebuur *et al.*, 2019 ▸; Kreuzer *et al.*, 2025 ▸). Thermal or hot neutron spectrometers, on the other hand, can detect a much broader energy range, typically with an improved resolution when the sample is cooled down to a few kelvin (Parker *et al.*, 2014 ▸). In soft-matter samples, mainly the vibrational dynamics of hydrogen atoms are probed.

Since neutron scattering is sensitive to nuclear displacements and not to changes in molecular polarizability like Raman spectroscopy, it is not subject to selection rules and all vibrational modes are active and measurable (Ramirez-Cuesta *et al.*, 2009 ▸; Kearley & Johnson, 2010 ▸). Therefore, QENS and Raman spectroscopy provide highly complementary data about diffusive motion, foremost that of hydrogen atoms and periodic vibrational dynamics. By measuring QENS and Raman simultaneously, a direct correlation between diffusional and vibrational properties (QENS) and the local chemical environment, meaning individual bond vibrations, molecular conformations and hydrogen-bonding interactions (Raman), is enabled under identical sample conditions (*e.g.* temperature, relative humidity *etc.*). This is particularly important for dynamic systems where environment-sensitive processes (such as hydration/dehydration, phase transitions or electrochemical cycling) influence both vibrational and diffusive behaviour. Such correlated observations can help to disentangle coupled phenomena (*e.g.* mobility changes associated with structural rearrangements). Until now and to the best of our knowledge, very few efforts have been made to integrate QENS and Raman in combination with environmental control. A dedicated Raman setup is available on the TOSCA instrument at the ISIS Neutron and Muon Source (UK), where it has been combined *in situ* with neutron vibrational spectroscopy (Adams *et al.*, 2009 ▸), albeit without control of the relative humidity (RH). The combination is achieved inside a cryostat for low temperatures, as is typically required for vibrational spectroscopy on the neutron energy loss side, using thermal or hot incident neutrons.

To perform an accurate measurement, independent of the technique used, control over environmental parameters such as temperature, ambient RH and composition of the surrounding vapour is crucial as they can affect the behaviour of soft-matter systems significantly (Kim & Matsunaga, 2017 ▸; Kreuzer *et al.*, 2021*c* ▸; Qian *et al.*, 2024 ▸). Literature reports have been published on numerous humidity chambers that are used to control RH and the composition of the vapour surrounding the sample. For this purpose, saturated salt solutions (Young, 1967 ▸; Carotenuto & Dell’Isola, 1996 ▸), heated solvent reservoirs (Bießmann *et al.*, 2018 ▸; Kreuzer *et al.*, 2019 ▸) or solvent-saturated gas flows are typically used (Thijs *et al.*, 2007 ▸; Oerter *et al.*, 2019 ▸). The described chambers are usually small, allowing for fast humidity (and temperature) switches but limiting the sample size and available space around the sample, which could be occupied by additional (smaller) measurement equipment (*e.g.* for four-point conductivity measurements), sensors (RH, temperature or gas), external stimuli (UV/IR light), or different sample holders for solutions, films and powders.

Here, we present a newly developed dedicated sample environment (SE) enabling simultaneous QENS and Raman measurements on different sample geometries. The feasibility of the SE is demonstrated on polymer films under controlled and variable RH and temperature. At present a sample holder is available for self-standing or substrate-supported polymer films. The SE consists of several components: a spherical 3D-printed measurement chamber, in which the polymer films (freestanding or on a suitable substrate) are installed, a Raman spectrometer, and a custom-built gas-flow setup and thermostat for control over RH (5–85%) and temperature (20–50°C). The measurement chamber provides sufficient space for large samples (5 × 5 cm) and additional measurement equipment, while the custom-built gas-flow system and embedded heating/cooling channels within the chamber walls allow for quick changes in RH and temperature.

**Figure 1:**
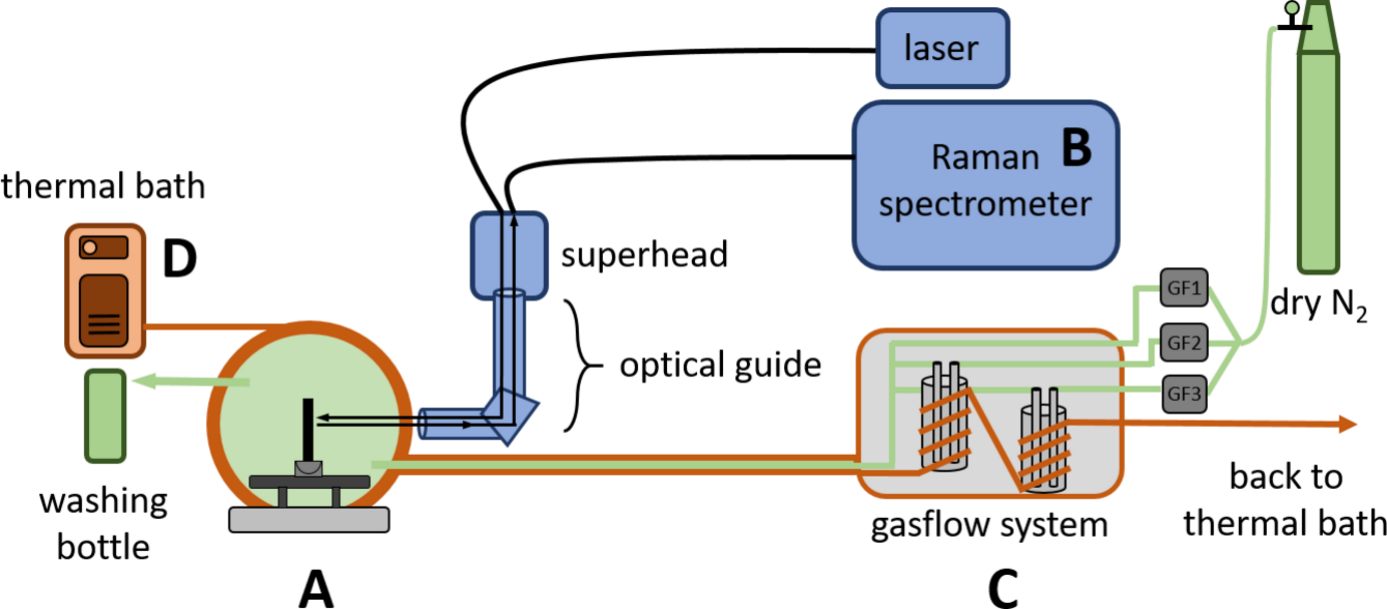
Scheme of the entire setup with the four main overall components: the measurement chamber (A), Raman spectrometer and equipment (B, blue), gas-flow system (C, green) and thermal bath (D, red). The working principle of each component and how they are connected to each other are described in the main text. The neutrons enter the chamber with an angle of 45° with respect to the laser light.

A schematic overview of the entire setup is shown in Fig. 1 ▸. The modular approach enables a quick and flexible assembly of the entire setup at various neutron sources and therefore provides a reliable platform for simultaneous QENS and Raman measurements on various soft-matter systems, such as polymer films, under variable RH and temperature.

In the first part of this article, we will describe the developed QENS chamber and its control over RH and temperature. Then in the second part we will show data from simultaneous QENS and Raman measurements on poly(3,4-ethylene dioxy­thiophene):poly(styrenesulfonate) (PEDOT:PSS) films on an Si substrate. PEDOT:PSS is a well studied polymer blend and serves here as a model system (Fan *et al.*, 2019 ▸; Yang *et al.*, 2020 ▸; Kong *et al.*, 2022 ▸).

## 2. Experimental section

### 2.1. Materials

PEDOT:PSS (high-conductivity grade, resistance < 100 Ω/sq,refractive index = 1.334, density = 0.999 g ml^−1^, 1.1 wt% aqueousdispersion, surfactant free) was purchased from Sigma–Aldrich and used as received. De-ionized H_2_O was used from a MilliQ setup (resistance of 18.2 MΩ).

### 2.2. Film preparation: spray-deposition of PEDOT:PSS on an Si substrate

The PEDOT:PSS solution was mixed with H_2_O (volume ratio of 1:1, final concentration of PEDOT:PSS 0.55 wt%) and stirred overnight using a magnetic stirrer bar. PEDOT:PSS films were prepared by spraying the aqueous PEDOT:PSS solution layer by layer on a heated substrate (120°C) using a static nozzle (Compact JAU D555000, Spraying Systems Co., Belgium). Silicon wafers (thickness of 525 ± 25 µm) were used as substrates (one side polished, Si-Mat, Germany). The distance between the nozzle and substrate was 20 cm. The first 20 single layers were sprayed with 200 ms spray pulses at a flow rate of 6.5 l min^−1^. After that, 60 spray pulses, each taking 200 ms, were sprayed on top at a flow rate of 7.5 l min^−1^. The initially lower flow rate is necessary to ensure the first layers have high homogeneity. The drying time between spraying the individual layers was 30 s. The final film thickness was around 10 µm.

### 2.3. Neutron scattering

Quasi-elastic neutron scattering (QENS) experiments were performed on the cold neutron time-of-flight spectrometer FOCUS at the Paul Scherrer Institut (PSI) in Villigen, Switzerland. In order to install the measurement chamber on FOCUS, an adapter plate was designed, which was mounted directly on the sample table of FOCUS. The chamber itself was then screwed to this adapter plate and connected to the Raman instrument, gas-flow system, thermal bath and RH/temperature sensor. A radial collimator was used to reduce the background scattering from the sample environment setup. Additionally, the chamber was temporarily shielded with boron to avoid potential scattering from any of the chamber components. Nevertheless, some small peaks caused by scattering at the chamber wall are still visible, at a shifted time, corresponding to the change in flight path. These peaks do not affect the signal and analysis of the quasi-elastic regime. The background scattering based on the relative humidity inside the chamber is very low, as shown later in this paper in Fig. 7.

**Figure 2:**
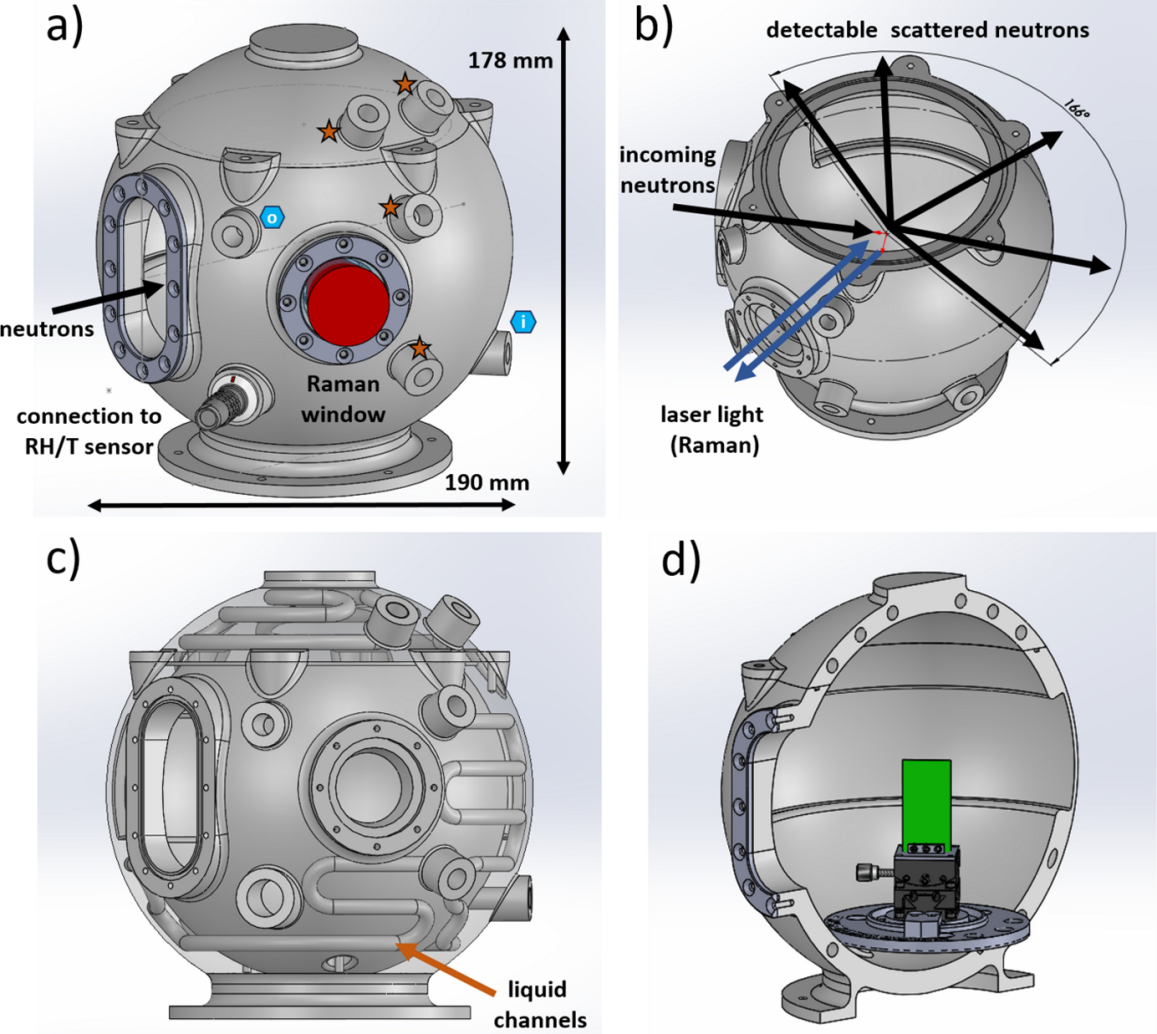
(*a*) Side view showing the neutron and Raman entry window. The Raman window also serves as an exit window for the reflected Raman light. The red cone indicates the expanded laser light. Orange stars and blue hexagons mark connections for the thermal bath and gas-flow system (‘i’ for in and ‘o’ for out mark the flow direction). A LEMO plug can be used to bring any electronics, *e.g.* RH/*T* sensors or additional measurement equipment, inside the chamber. (*b*) The scattered neutrons exit through the chamber wall. Over an angular area of 166° and 4 cm in height, the chamber wall has a thickness of only 2 mm. (*c*) The thicker chamber walls host liquid channels, which can be used to cool and heat the inside of the chamber by connecting it to a thermal bath. (*d*) Chamber with sample holder and sample (indicated in green).

The measurements were performed with an incoming neutron wavelength of 5.8 Å, which results in an instrumental resolution of 45 µeV (full width at half-maximum). The resolution was obtained by measuring a vanadium sample, which scatters purely elastically and features no internal dynamics. The highest accessible momentum transfer *q* using this configuration is *q* = 2.0 Å^−1^. The measurement times were 8 and 6 h for the PEDOT:PSS film in dry and humid environments (at 25 and 50°C), respectively. The relatively long measurement time was mainly due to the low sample thickness of 10 µm, which we consider to be the minimal thickness for PEDOT:PSS samples for QENS measurements on FOCUS. The Si substrate (thickness of 525 µm) with the PEDOT:PSS film (thickness of 10 µm) on top was fixed to an aluminium plate (thickness of 1 mm), and this plate was installed on the custom-designed sample holder by means of three screws [Fig. 2(*d*)]. The data were processed, visualized and analysed using the software package *DAVE* (*Data Analysis and Visualization Environment*; Azuah, 2009 ▸).

### 2.4. Raman spectroscopy

For Raman measurements a Horiba iHR 320 spectrometer equipped with a CCD detector, light source, automated shutter control, superhead, optical guide and optical fibres was used. The connection of these components is shown schematically in Fig. 1 ▸ (component B). To avoid any sample degradation due to strong laser radiation, the laser power is attenuated and the attenuated laser light (λ = 785 nm) hits the sample with approximately 7 mW. The entire laser pathway is shielded. To reduce the risk of laser exposure further, the Raman shutter was manually closed and the laser switched off before the measurement chamber was approached.

The laser light is guided via optical fibres, first to the superhead and optical guide before then reaching the sample. The purpose of the superhead is to deliver the laser light to the sample efficiently and to collect and filter the returning Raman signal (via a set of Rayleigh and interference filters). The optical guide expands the laser beam to a width of approximately 30 mm, before the widened laser light passes through a final lens within the optical guide. This lens exhibits a focal length that precisely matches the distance to the sample (100 mm). Thus, the focus spot of the laser light is on the sample, which optimizes the obtained Raman intensity. To check for potential laser radiation damage and to analyse the heat load, the PEDOT:PSS sample was exposed to continuous 1 h Raman measurements (for 8 h overall) under constant RH and temperature. No changes were observed between the first and last Raman spectra, which confirmed no radiation damage, while the heat load can also be neglected.

The reflected Raman signal travels back the way it came: first through the optical system and superhead. The Raman signal is then guided to the Raman spectrometer and CCD detector via optical fibres. The spectrometer features three different gratings (600, 1200 and 1800 lines mm^−1^). In addition, three different measurement modes are available: best dynamic range, intensive light and high sensitivity. For polymer films, the best Raman spectra are typically recorded using the high-sensitivity mode. The Raman spectrometer was placed on top of the FOCUS instrument and shielded with borated polyethylene sheets to protect the CCD detector from scattered neutrons and γ-radiation. Spectra were recorded within a wavenumber range of 400 to 1600 cm^−1^ with a measurement time of 1 h per spectrum. For data analysis, the individual spectra (overall eight spectra for the PEDOT:PSS film in a dry environment and six spectra for the film in a humid environment at 25 and 50°C) were binned together.

## 3. Results

### 3.1. Three-dimensionally printed measurement chamber

The design of the QENS chamber is shown in Fig. 2 ▸. It is entirely 3D-printed via selective laser melting (EOS M290, PROTIQ GmbH, Blomberg, Germany) from the aluminium-based alloy AlSi10Mg. The printing process takes less than 24 h and thus allows for rapid chamber modification or duplication. Note that the version described here is based on a previously reported chamber, which was initially designed for neutron reflectometry and (grazing-incidence) small-angle neutron scattering (Widmann *et al.*, 2020 ▸; Widmann *et al.*, 2021 ▸; Kreuzer *et al.*, 2021*a* ▸; Kreuzer *et al.*, 2021*c* ▸; Le Dû *et al.*, 2025 ▸).

The version presented here has been modified for QENS experiments with the possibility of simultaneous Raman measurements. Neutrons enter the chamber through a window and hit the sample, which is mounted at the centre of the chamber. The entry window is made of Al foil (thickness, width and height of 0.1, 30 and 68 mm, respectively), which is fixed to the chamber via an Al frame [Fig. 2 ▸(*a*)] and 12 screws (made from Al). The scattered neutrons exit the chamber directly through the 3D-printed chamber walls. To maximize the number of detectable scattered neutrons and reduce the background, a large area of the chamber wall (approximately 5 cm in height over an angular range of 166°) was printed with a thickness of only 2 mm. Fig. 2 ▸(*b*) indicates the neutron exit window and highlights the pathway of the incoming and scattered neutrons (black arrows). The (in­coherently) scattered neutrons are attenuated by the neutron exit window by less than 1%, as calculated using the neutron attenuation equation *I* = *I*_0_ exp(−Σ*t*), with *I*, *I*_0_, Σ and *t* being the transmitted neutron intensity after leaving the neutron window, the initial neutron intensity, the macroscopic absorption cross section and the thickness of the neutron window, respectively.

The Raman window is located next to the neutron entry window [Fig. 2 ▸(*a*)]. It is made from uncoated λ/10 fused silica, which is transparent to the Raman laser light within a wavelength range of 400 to 2200 nm and has a diameter of 32 mm. The distance between the Raman window and the sample is 88 mm. After being reflected by the sample, the laser light exits the chamber through the same window [blue arrows in Fig. 2 ▸(*b*)]. A custom-built optical guide and a superhead guide the Raman signal to the Raman spectrometer and the detector. This is described in more detail in Section 2.4.

Our setup is able to provide stable environmental conditions with respect to RH and temperature. A gas-flow system transports a constant flow of dry, humidified, or a mixture of dry and humidified nitrogen gas into and out of the chamber. Fig. 2 ▸(*a*) indicates the gas inlet and outlet with blue hexagons. The working principle of the custom-built gas-flow setup is provided in Section 3.2. Having a continuous flow of controlled humidity through the measurement chamber ensures a stable and homogeneously distributed RH inside the chamber, thereby preventing any fluctuations in neutron scattering due to varying water vapour concentrations, *e.g.* because of temperature gradients. Moreover, the spherical shape of the measurement chamber and the minimization of any edges and corners inside the chamber reduce condensation and thus allow for stable and hours-long high-humidity experiments. This is especially beneficial for neutron scattering experiments, which often require long measurement times, in particular when studying thin polymer films.

The 3D-printing technique allows channels to be embedded within the chamber walls, through which a cooling or heating liquid flows for temperature control [Fig. 2 ▸(*c*)]. This supports a homogeneous temperature distribution inside the chamber, even though the neutron exit window, where no channels are embedded, makes up a large area of the chamber walls and thus an asymmetric distribution of these channels is present. A more detailed description and temperature simulation are described in the latter part of this section. With the large size of the chamber (inner diameter of 150 mm) and the vertical sample geometry, the distance of a typical sample for QENS [2 × 4 cm in width and height; Fig. 2 ▸(*d*)] from the chamber walls (*i.e.* the cooling/heating channels) is at least 65 mm in both horizontal and vertical directions. This minimizes the temperature gradient at the sample, which is due to the asymmetric cooling/heating channel distribution inside the chamber walls. On the other hand, because of the large chamber size and volume, it takes a longer time (on the scale of minutes) to change the RH and temperature. Since QENS measurements on polymer films are typically in the range of several hours this is rarely an issue. Additionally, the large chamber size allows the installation of further equipment, such as additional (smaller) measurement equipment (*e.g.* for four-point conductivity measurements), sensors (RH, temperature or gas), external stimuli (UV/IR light), or different sample holders for solutions, films and powders.

**Figure 3:**
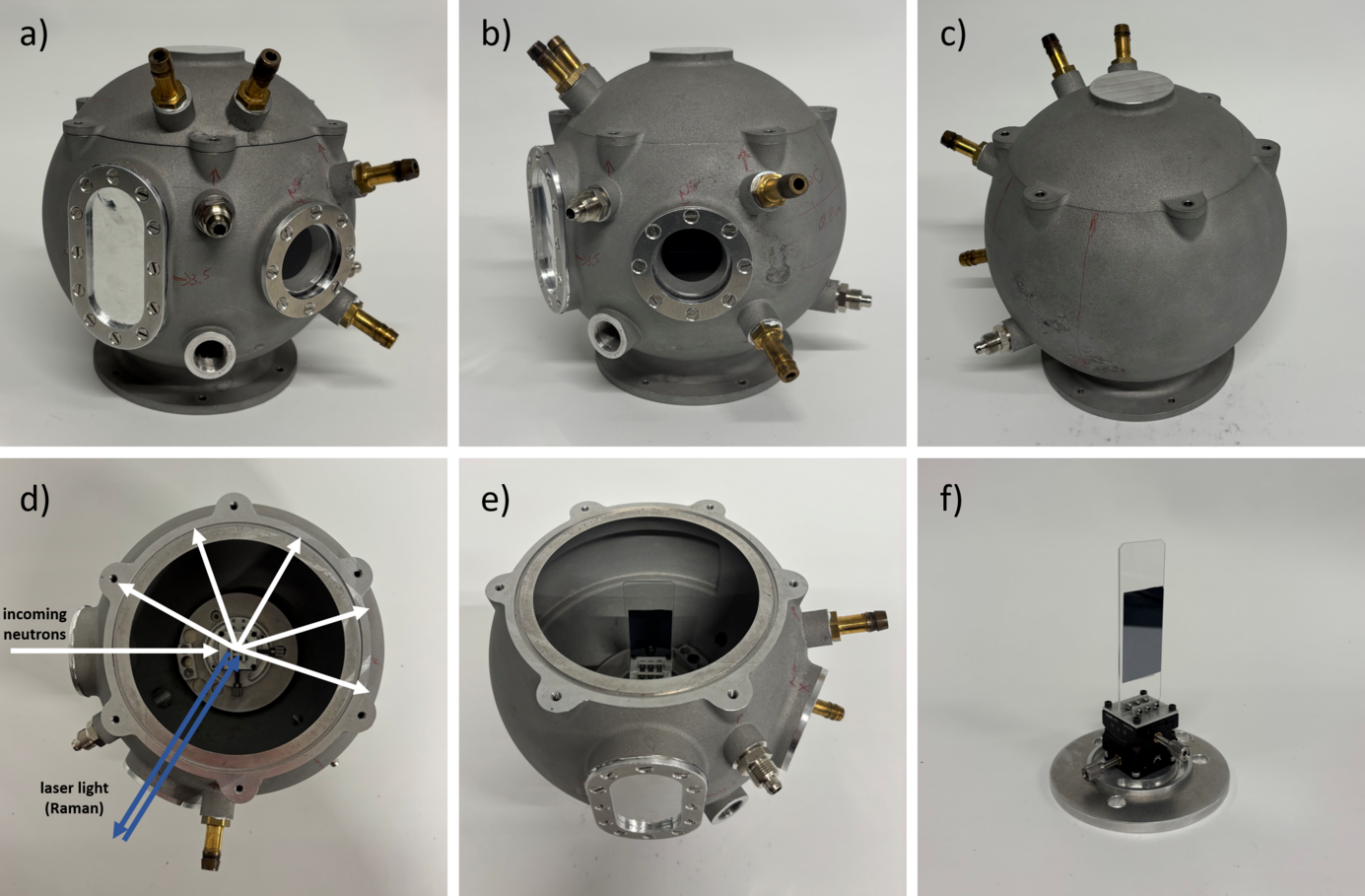
Photographs of the 3D-printed measurement chamber, (*a*)–(*c*) from different angles, (*d*) from the top including the neutron (white) and laser light (blue) pathways, (*e*) from diagonally above, where the neutron exit window is visible, and (*f*) of the sample holder with an Si wafer fixed to a thin plastic plate. For simultaneous QENS and Raman measurements, the plastic plate was replaced by an aluminium plate to minimize the scattering background. The PEDOT:PSS film on the Si substrate was fixed to this aluminium plate by means of aluminium wire.

Fig. 3 ▸ provides some images of the 3D-printed chamber. The sample holder displayed in Fig. 3 ▸(*f*) features two goniometers and can easily be modified for other sample geometries. Currently, we are putting together initial ideas for a designated sample holder for powders. The main challenge here is how to expose the powder homogeneously to a certain relative humidity without glueing it onto a substrate or imposing a gradient. For experiments on powders, and for liquids that do not involve a changing humidity, respective sample holders are already available.

**Figure 4:**
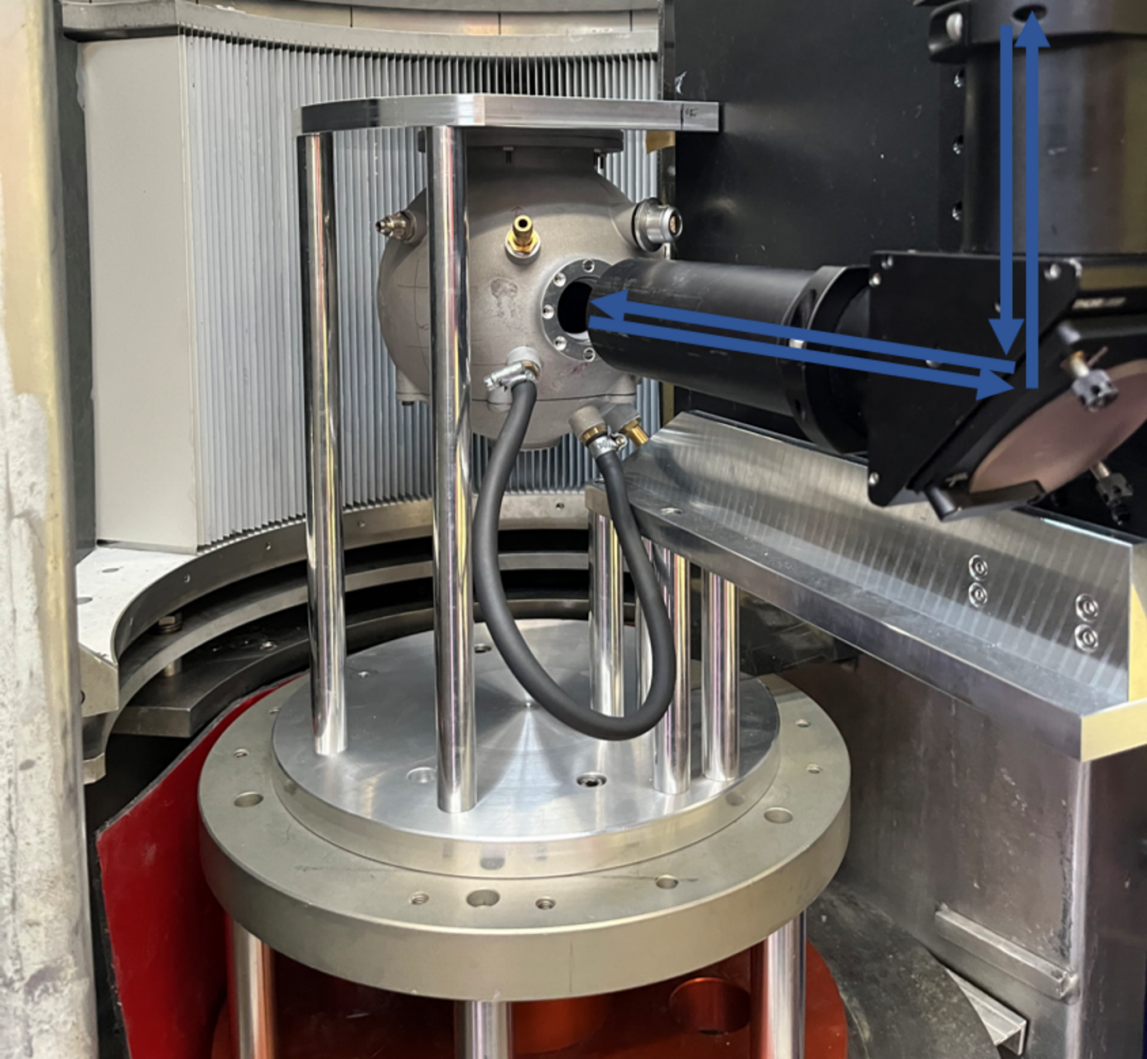
The measurement chamber mounted on the FOCUS instrument (Villigen, PSI). The pathway of the Raman laser and reflected signal through the optical guide is marked in blue.

Fig. 4 ▸ shows the chamber on the FOCUS instrument. The chamber is mounted upside down onto an adapter plate, since it was initially designed for the time-of-flight spectrometer TOFTOF at the neutron source FRM II in Munich, Germany, where the detector is on the other side compared with FOCUS. The pathway of the Raman laser and the reflected Raman signal is indicated in blue. The Raman spectrometer is placed on top of the FOCUS instrument. Generally, the setup can be installed on any QENS instrument, or more generally any neutron spectrometer and beyond, provided enough space is available. Note that the current setup is not designed for experiments under vacuum, so besides space requirements this is another limiting factor. In principle, the chamber could easily be re-designed to be vacuum tight to allow for a certain humidity inside the chamber. Alternatively, a container for the measurement chamber could be designed, so that the chamber inside the container would fit into a respective QENS instrument. However, both approaches would result in time-consuming sample changes, which is why they were not considered here.

### 3.2. Humidity and temperature control

The layout of the gas-flow system is shown in Fig. 1 ▸ (component C) and is described in detail by Widmann *et al.* (2021 ▸). In general, it consists of a constant supply of dry nitrogen, three remotely controlled gas-flow meters (GFs) including a power supply and control unit, two washing bottles to humidify the nitrogen stream (with two different solvents if needed), and the connection to the measurement chamber. The flow rate of the nitrogen is adjusted via the GFs, which are calibrated to a maximum flow of 1 l min^−1^ (at a working pressure of 2 bar). This pressure has proven to be suitable for fast humidity changes within the chamber, while keeping the nitrogen consumption reasonable (especially for beamtime sessions that take several days). Of the three GFs, one is used for dry nitrogen gas only (GF1 in Fig. 1 ▸), while the other two are connected to the two individual washing bottles (GF2 and GF3), where the nitrogen gas is humidified with desired solvents (*e.g.* water and organic solvent, or water and heavy water, which is particularly beneficial for neutron scattering, since it may selectively modulate the contribution of the exchangeable H atoms to the QENS signal). For experiments which require both dry and humid environments, using GF1 (dry nitrogen) and GF2 (humidified nitrogen) is sufficient. Since the gas flows through heated (or cooled) tubing maintained at the same temperature as the chamber, the effect of the gas flow entering the chamber is negligible.

To test the environmental control of RH and temperature inside the measurement chamber, nitrogen was flowed through one washing bottle filled with water with a stepwise increasing flow rate (from 0 to 100% in steps of 25%), while simultaneously the flow rate of nitrogen was decreased (from 100 to 0% in steps of 25%). The washing bottles are wrapped with hollow copper tubing to adjust the respective solvents to the desired temperature. Here, the temperature was kept constant at 20°C (for the washing bottles, tubing and chamber). The entire system is connected to a thermal bath, which cools or heats the measurement chamber first, before the cooling/heating liquid reaches the two washing bottles and the gas-transporting tubes (Fig. 1 ▸). This means the temperature of the entire setup is controlled by a single thermal bath, in which the dry or humid gas is slightly cooler than the measurement chamber (simply due to the flow direction of the thermal bath). Consequently, cooler gas flows into warmer surroundings in the chamber, which helps to avoid condensation. After a maximum RH has been reached inside the measurement chamber, we re-dried the chamber, increased the temperature to 50°C and repeated the above-described procedure.

**Figure 5:**
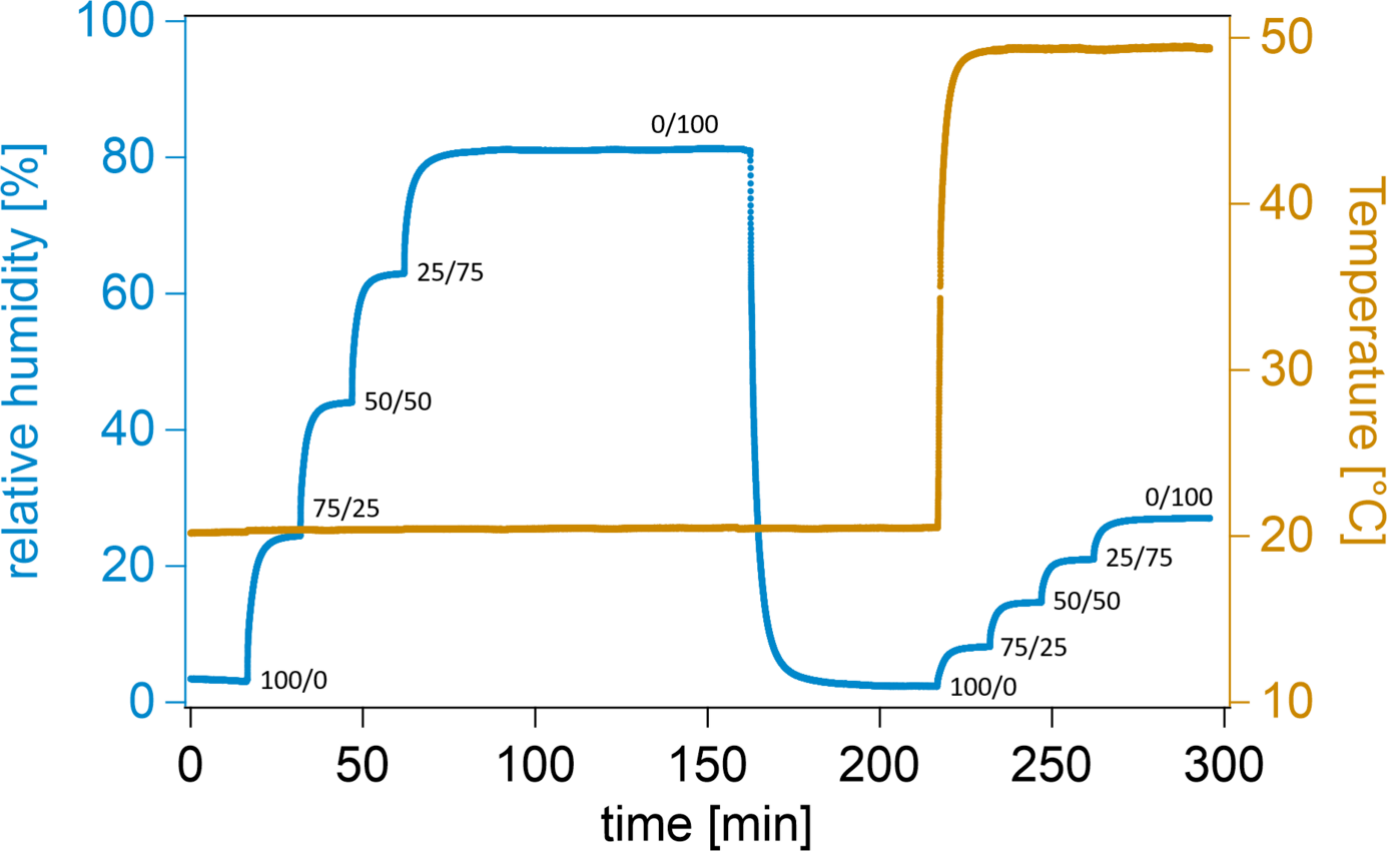
The relative humidity (blue) and temperature (orange) are measured at the centre of the measurement chamber and plotted as a function of time. The ratios of GF1 (dry) and GF2 (humid) are indicated at the respective humidity plateaux. The uncertainties in the RH and temperature are ±1.0%RH and ±0.1°C, respectively.

Fig. 5 ▸ shows the evolution of RH (blue) and temperature (orange) inside the chamber. The flow ratios of GF1/GF2 are marked next to the RH graph. The RH and temperature inside the measurement chamber are recorded with an RH/*T* sensor (Sensirion AG, Staefa, Switzerland). At 20°C, a large RH range from 3 to 80% can be covered, while at 50°C this range decreases to approximately 1.5 to 25%. This demonstrates how closely temperature and RH are coupled: at elevated temperatures, the surroundings can take up more water molecules and consequently the RH decreases, whereas the absolute number of water molecules in the gas phase remains constant. Note that strong changes in ambient temperature, *e.g.* in summer when it cools down overnight, might lead to higher uncertainty in the RH inside the chamber of approximately ±3%, despite the thermal control via the thermostat. This can become an issue for very long measurements running day and night, and for certain techniques like neutron diffraction, where the measured atomic distances can vary significantly with only slight changes in RH. However, for QENS measurements of a few hours, this is rarely a problem. The setup also allows for rapid RH and temperature switches (on the scale of a few minutes). In our tests, we were able to dry the chamber (from 81% to 10%) within 5 min and heat the chamber from 20°C to approximately 43°C within 3 min (Fig. 5 ▸). Note that the available temperature range for the setup is larger than the 20–50°C window described here. By using an oil-based heating liquid, temperatures of around 100°C can be reached inside the chamber. In previous tests at such temperatures, the gas-flow system was not connected to the chamber. Potentially, temperatures below 0°C are also possible, but this has not been tested so far.

The use of all three GFs opens up further possibilities. Especially interesting for studies that require different neutron contrasts is the use of H_2_O and D_2_O as individual solvents. In this way, the vapour composition around the sample can be adjusted to the desired H_2_O:D_2_O ratio, which matches the neutron scattering length density (SLD_N_) of parts of the sample and allows for contrast variation measurements. Organic solvents can be used as well, but it is important to install one-way gatekeepers between the washing bottles and the GFs, as the GFs might lose their calibration upon contact with organic solvents. A washing bottle installed after the measurement chamber helps to trap any organic solvents (Fig. 1 ▸). In recent studies, a combination of water and organic solvents (methanol and acetone) has been used to investigate co-nonsolvency effects in polymer thin films in mixed vapours with neutron reflectometry (Kreuzer *et al.*, 2021*a* ▸; Kreuzer *et al.*, 2021*b* ▸; Geiger *et al.*, 2021 ▸; Wang *et al.*, 2022 ▸). For a desired ratio of two solvents in the vapour phase, the respective vapour pressures have to be considered, especially when an organic solvent and water are used, as their vapour pressures differ significantly. A good starting point in calculating such ratios is the Antoine equation and Raoult’s law.

**Figure 6:**
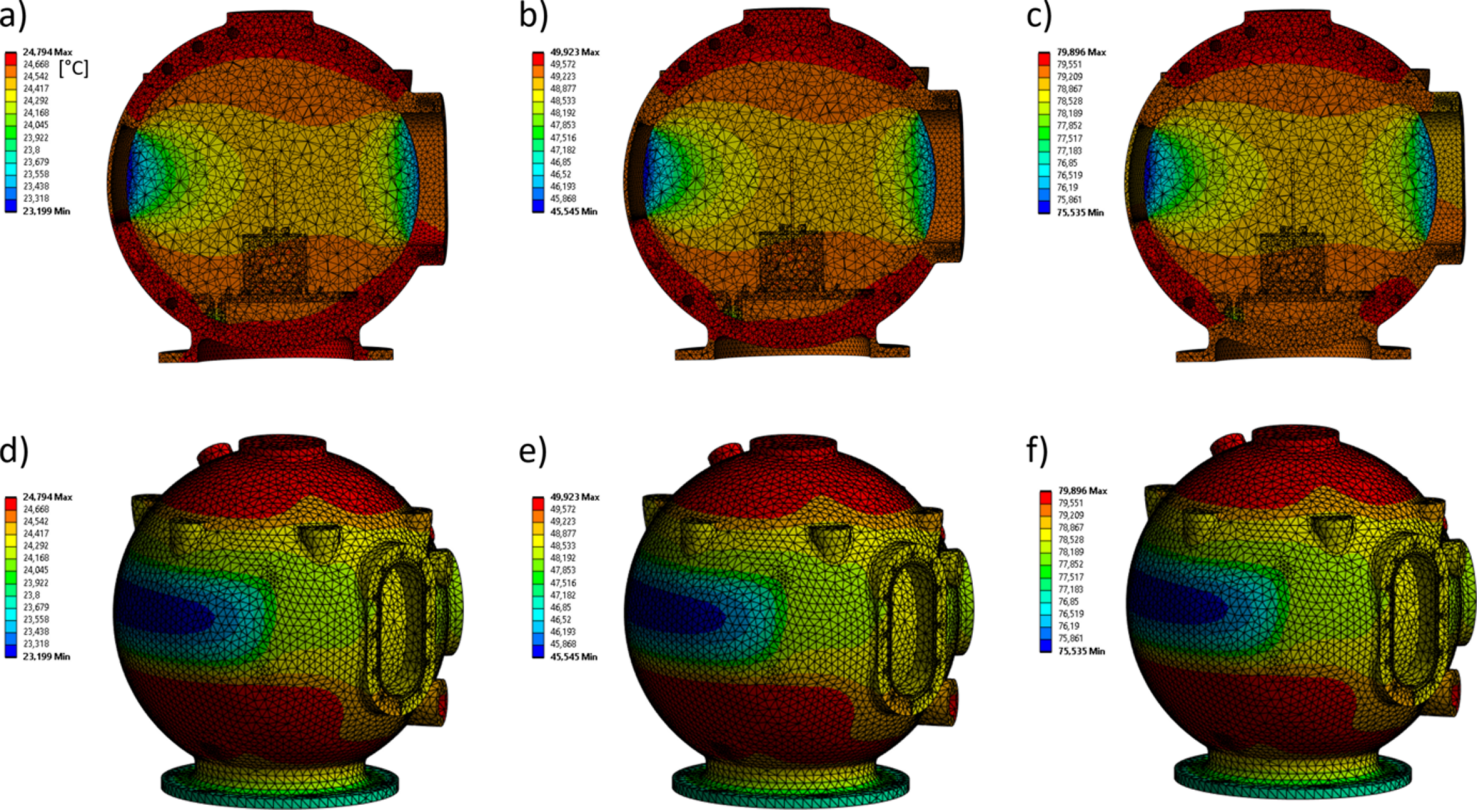
Simulations of the temperature distribution inside the QENS chamber (top row) and of the outer walls (bottom row) at (*a*), (*d*) 25°C, (*b*), (*e*) 50°C and (*c*), (*f*) 80°C. All temperature values in the figure are given in °C.

In addition to the laboratory experiments shown in Fig. 5 ▸, we simulated the temperature distribution at different tem­peratures (25, 50 and 80°C) inside the measurement chamber. The simulations were done using the computational fluid dynamics software of Ansys Inc. (*Ansys Fluent*; https://www.ansys.com/products/fluids/ansys-fluent) and take into account a nitrogen flow of 1 l min^−1^ through the chamber. The ob­tained temperature distributions inside the QENS chamber and of the outer walls are shown in Fig. 6 ▸. Despite the large neutron exit window, where no heating/cooling channels are embedded, the simulations indicate a homogeneous tem­per­ature distribution at the sample position for all three simulated temperatures. The simulated temperatures agree well with the experimentally measured ones; *e.g.* with the thermostat set to 50°C, the temperature at the sample position is 48.8°C (simulation) and 49.0°C (temperature sensor).

To optimize the described setup further, an updated version of the gas-flow and thermostat system is under construction, which features improved temperature control (*e.g.* by reducing the lengths of the connection tubes and integrating an electric heater), smaller washing bottles and thus a decreased solvent volume, a feedback system that couples the measured RH inside the chamber with the gas-flow rate through the GFs, and an overall higher total flow through the GFs. Thereby, we aim to increase the available range of relative humidities inside the QENS chamber at different temperatures and further decrease *e.g.* the drying times of the humidified chamber via the dry nitrogen flow (ideally to a few minutes) to reduce waiting times during neutron beamtime sessions.

### 3.3. Simultaneous QENS and Raman measurements on PEDOT:PSS films

**Figure 7:**
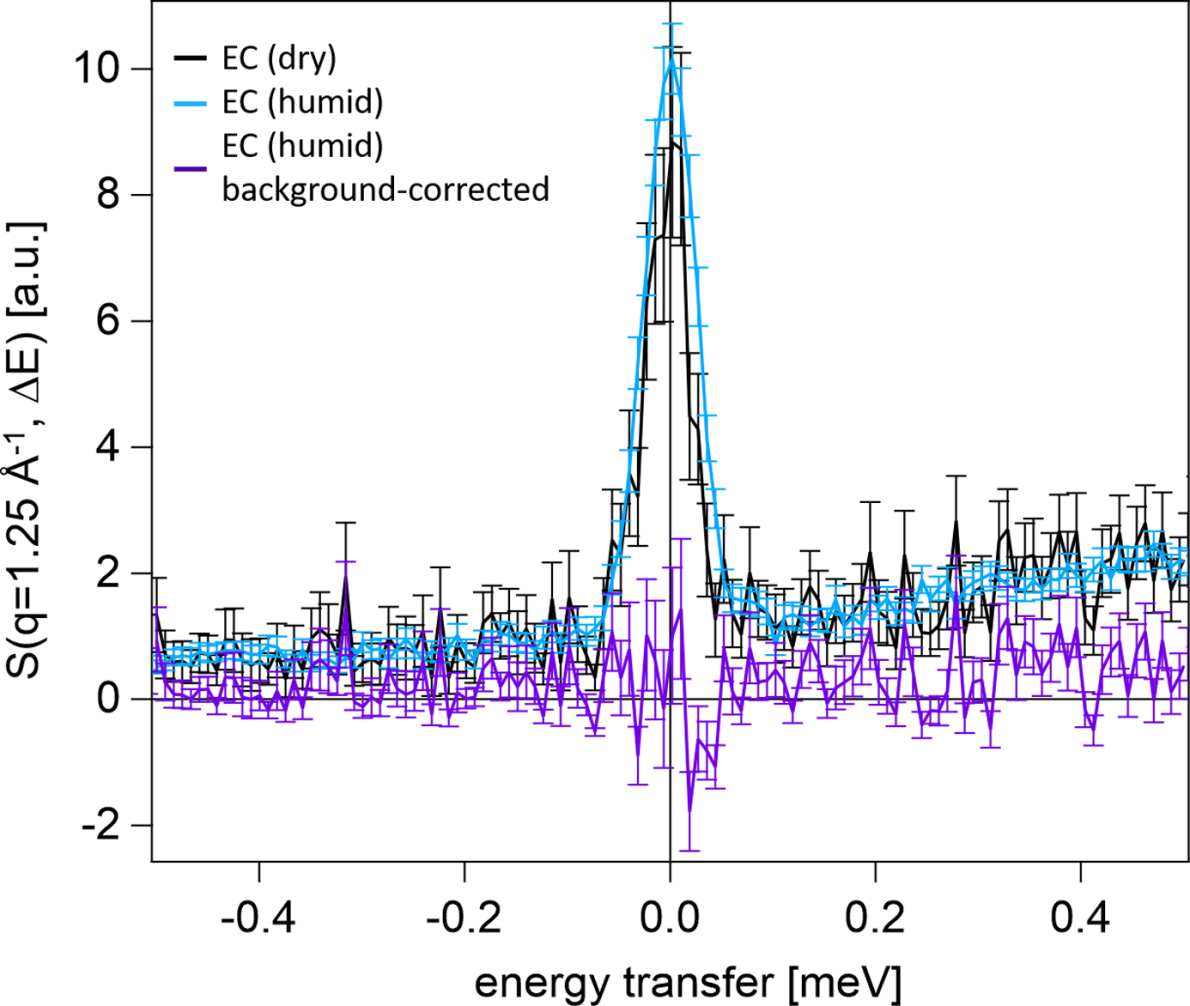
QENS signal of the empty measurement chamber in a dry (RH = 5%, black) environment. The signal stems from incoherent scattering at the aluminium walls of the chamber and is referred to as background signal herein. The increasing scattering intensity on the positive energy transfer side can be ascribed to the time-independent background signal of the FOCUS instrument. In addition, the signal of the empty measurement chamber in a humid (RH = 82%) environment is shown without (blue) and with (purple) background correction. For clarity, the zero lines for both axes are plotted as well.

QENS measurements were performed on the time-of-flight spectrometer FOCUS at the Paul-Scherrer Institute in Villigen, Switzerland. The modular approach of the above-described components, *i.e.* measurement chamber, Raman spectrometer, gas-flow setup and thermostat, allowed for quick assembly on the FOCUS instrument in approximately 3 h. After the alignment of the measurement chamber, we first obtained the instrument resolution by measuring a vanadium plate. The plate was of the same dimensions as the actual samples (PEDOT:PSS film on a 1 mm thick Si substrate, 2 × 4 cm). To determine the scattering background of the chamber, we removed the vanadium plate and measured the empty chamber in a dry environment (RH = 5%). The obtained dynamic structure factor *S*(*q* = 1.25 Å^−1^, Δ*E*) is shown in Fig. 7 ▸ and is considered as background for all further measurements. The use of a radial collimator significantly reduces the background scattering from the sample environment (including the air inside the measurement chamber). Nevertheless, small peaks caused by scattering at the chamber wall are still visible, albeit at a shifted time corresponding to the change in flight path and thus not affecting the signal and analysis of the quasi-elastic regime. In addition, we carried out a QENS measurement of the empty chamber in a humid environment (RH = 82%). The respective dynamic structure factor, with and without background correction, is also plotted in Fig. 7 ▸. No additional signal around the elastic line is visible in the uncorrected measurement (blue line), which demonstrates that the gaseous water molecules of the humid vapour inside the chamber are not affecting the QENS measurements of the PEDOT:PSS films. Consequently, the corrected empty-chamber measurement (purple line) shows zero intensity throughout the entire energy-transfer spectrum. Only a scaling factor was used to correct the increasing neutron absorption at high humidities. Altogether, these measurements ensure that the measurement chamber provides an easy-to-install environment with low scattering background for QENS measurements. As we had an open sample environment space on FOCUS, background scattering might even be further reduced by an optimized shielding, a neutron guide or vacuum tube up to the measurement chamber, and the usage of argon or helium inside the chamber (instead of air).

**Figure 8:**
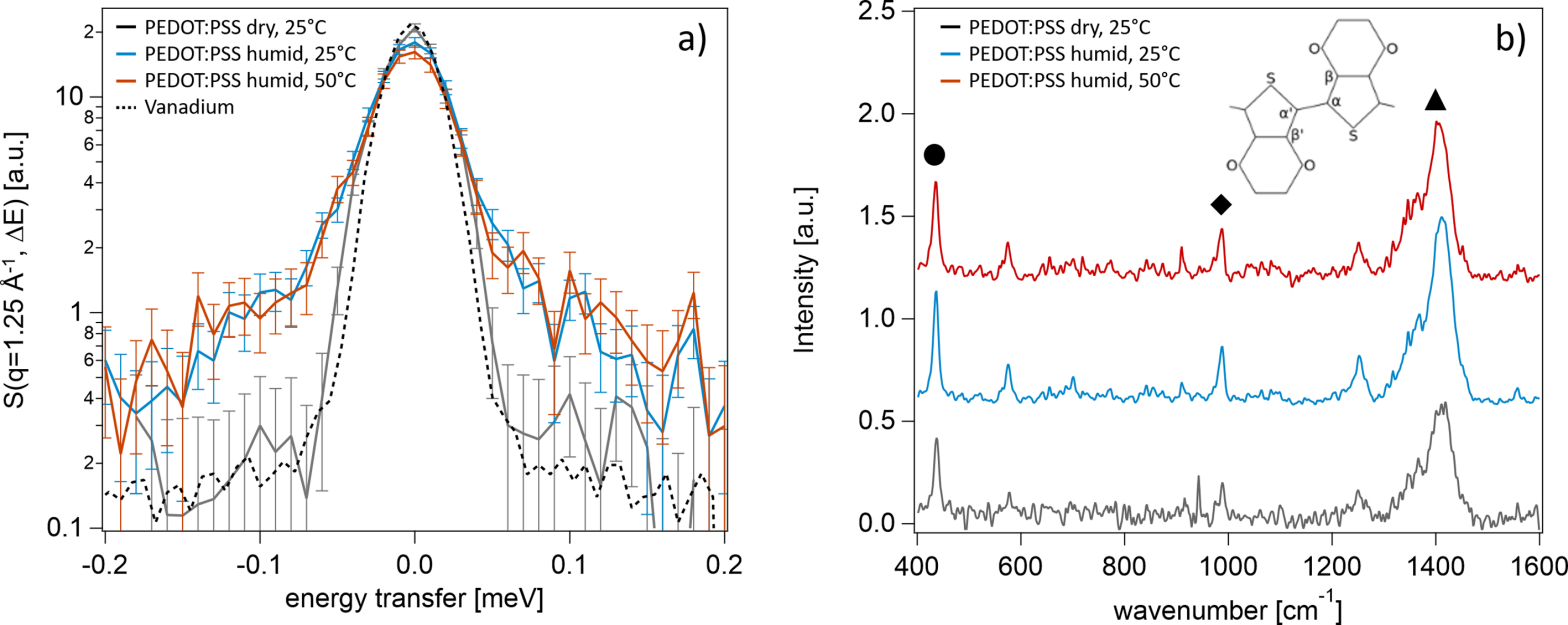
(*a*) QENS and (*b*) Raman spectra of PEDOT:PSS films at 25°C in dry (grey) and humid (blue) environments (5 and 82%, respectively) and at 50°C in a humid (26%) environment (red). The dynamic structure factors *S*(*q*, *E*) correspond to a momentum transfer of *q* = 1.25 Å^−1^. The black diamond and triangle indicate the peaks that refer to the Cα—Cα′ (inter-ring) stretching and symmetric Cα=Cβ—(O) stretching, respectively, while the black sphere indicates the peak at 437 cm^−1^ as explained in the text. The chemical structure of PEDOT is shown as an inset in panel (*b*).

For simultaneous QENS and Raman measurements, the Si wafer with the PEDOT:PSS film on top was mounted at the centre of the measurement chamber. Overall, three measurements were performed, with the PEDOT:PSS sample in (i) a dry environment and (ii) humid water vapour (RH = 5 and 82%, respectively) at 25°C, and (iii) a humid environment (RH = 26%) at 50°C. Simultaneously, Raman spectra were recorded. The obtained dynamic structure factors *S*(*q*, *E*) (at a momentum transfer of *q* = 1.25 Å^−1^) and Raman data are shown in Figs. 8 ▸(*a*) and 8 ▸(*b*), respectively.

The dynamic structure factor of the dry PEDOT:PSS film at 25°C, shown in Fig. 8 ▸(*a*), exhibits only a slight increase in intensity around the elastic line compared with the vanadium measurement (black dashed line), while the overall scattering intensity remains low. This finding can be attributed to the thin-film thickness [10 µm in ambient conditions, *i.e.* RH of around 40%; in humid environments the thickness increases to around 20 µm (Bießmann *et al.*, 2018 ▸)]. Additionally, the absence of a quasi-elastic scattering signal suggests that no significant dynamics occur within this energy range. Apparently, no or very little water (or immobilized water featuring very slow dynamics) remains inside the PEDOT:PSS films in a dry environment. Polymer dynamics, originating from the PEDOT:PSS polymer chains and side groups, typically appear beyond the resolution limit of the instrument (Ono & Shikata, 2007 ▸; Rubio Retama *et al.*, 2008 ▸; Kreuzer *et al.*, 2025 ▸).

In contrast to the dry PEDOT:PSS film, a significant QENS signal is visible at small energy transfers [Fig. 8 ▸(*a*)] for the films in humid environments at 25 and 50°C. We ascribe this signal mainly to the dynamics of water molecules within the PEDOT:PSS film, because on the one hand we know that water is present within PEDOT:PSS films in humid environments (water content > 50 vol.%; Bießmann *et al.*, 2018 ▸), while on the other hand, in a previous QENS study, water dynamics in a PEDOT:PSS/cellulose composite film were found within the same energy range (Kreuzer *et al.*, 2025 ▸). The measurements at 25 and 50°C are very similar. It seems that temperature plays a minor role for the water dynamics, but it should be noted that water acts as a plasticizer and, in particular at higher temperatures, slower polymer dynamics (*e.g.* of the PEDOT:PSS side chains) might contribute to the detected QENS signal. To verify whether the QENS signal refers to polymer dynamics as well, reference experiments in humid D_2_O vapour have to be done. In these experiments, possible H/D exchange reactions in the polymer should be kept in mind and can be followed via Raman spectroscopy. For example, the Raman signal of an O—H bond appears at a Raman shift of 3000–3800 cm^−1^, while with O—D the signal would shift to lower wavenumbers.

**Figure 9:**
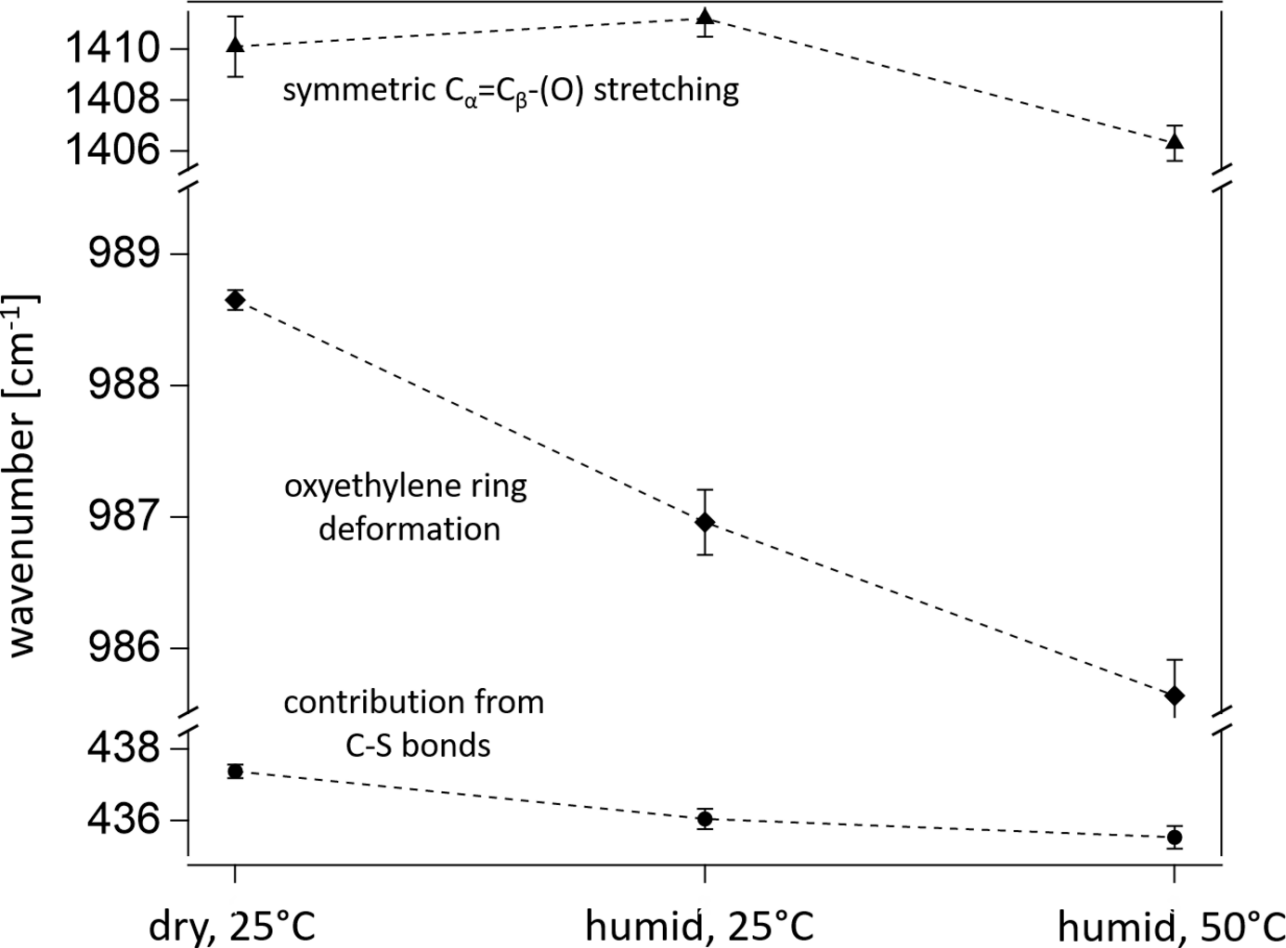
Shifts of the Raman peaks, corresponding to the deformation of the oxyethylene ring (bottom, centre) and stretching of Cα=Cβ—(O) (top) obtained via Raman spectroscopy.

Fig. 8 ▸(*b*) shows the corresponding Raman spectra that were obtained simultaneously with the QENS measurements. For all three measurements, we observe clear peaks that can be assigned to characteristic vibrations of especially PEDOT. The chemical structure of PEDOT is shown as an inset in Fig. 8 ▸(*b*), with the relevant C atoms labelled accordingly. In agreement with the literature, the most prominent signals appear at 437 cm^−1^ [contributions from the C—S bond, marked with a black sphere in Fig. 8 ▸(*b*)], 575 and 986 cm^−1^ [both referring to oxyethylene ring deformation; the latter is marked with a black diamond in Fig. 8 ▸(*b*)], 1250 cm^−1^ [Cα—Cα′ (inter-ring) stretching], and 1411 cm^−1^ [symmetric Cα=Cβ—(O) stretching, marked with a black triangle in Fig. 8 ▸(*b*)] (Garreau *et al.*, 1999 ▸; Chiu *et al.*, 2006 ▸; Nešpůrek *et al.*, 2021 ▸; Kong *et al.*, 2022 ▸). The Raman spectrum of the dry PEDOT:PSS film (grey curve) exhibits a slightly lower signal-to-noise ratio. This behaviour is due to a misalignment of the PEDOT:PSS film inside the measurement chamber, which reduced the area of the sample in the focus spot of the laser light. It was corrected for the subsequent measurements in humid environments. The QENS measurements were not affected by the misalignment, as the area of the sample illuminated by neutrons is significantly larger. Upon humidification and temperature change, some of the peaks shift, as can be seen in Fig. 9 ▸ for the three most prominent peaks observed at 437 cm^−1^ (contributions from the C—S bond), 986 cm^−1^ (oxyethylene ring deformation) and 1411 cm^−1^ [symmetric Cα=Cβ—(O) stretching].

The observed peak shifts indicate a change in the vibrational energy of the respective bonds. During humidification, this is due to altered hydration of the respective groups, meaning the dynamic structure of the hydration layer changes as water is absorbed into the PEDOT:PSS film, which consequently affects the vibrational bonding energies. This agrees with the QENS data, which confirm the presence of water dynamics in the film in a humid environment. Upon temperature increase, we see a further shift of the peaks obtained via Raman, whereas the QENS data feature no significant differences in *S*(*q*, *E*) of the humid films at 25 and 50°C. Apparently, temperature has only a weak effect on the average water dynamics (seen via QENS), while it affects the vibrational bond energies of the respective PEDOT groups, *e.g.* the contributions of the C—S bonds, the bonds in the oxyethylene ring and the Cα=Cβ—(O) bond (via Raman). These results highlight on the one hand the complementary information obtained by QENS and Raman spectroscopy. On the other hand they demonstrate that our setup is capable of detecting water (and potentially polymer) dynamics, as well as changes in the hydration behaviour of a PEDOT:PSS film and its functional groups under controlled and variable RH and temperature.

In summary, the possibility of performing both QENS and Raman simultaneously is very valuable, especially for complicated or not perfectly reproducible measurement protocols and costly samples.

## 4. Conclusions

We have developed a dedicated sample environment that enables simultaneous quasi-elastic neutron scattering and Raman spectroscopy experiments on polymer films under controlled and variable relative humidity and temperature. This sample environment consists of a spherical 3D-printed measurement chamber, which is connected to a custom-built gas-flow system and thermostat for precise environmental control. Laboratory tests confirm that the RH can be reliably and rapidly adjusted between 3% and 80% at 25°C, while integrated cooling and heating channels ensure stable and homogeneous temperature distribution. The chamber is made from an AlSiMg alloy, with a thin-walled neutron exit section, minimizing neutron absorption to below 1%. Empty chamber measurements verified that no significant background or secondary scattering arises from either the chamber itself or water vapour under humid conditions.

Simultaneous QENS and Raman measurements conducted on the time-of-flight spectrometer FOCUS (PSI, Switzerland) demonstrate the complementary nature of these techniques in studying polymer hydration and water dynamics. QENS successfully detected the presence and mobility of absorbed water molecules (and possibly fast polymer side-chain dynamics, especially at higher temperatures), while Raman spectroscopy provided insight into the vibrational modes of PEDOT:PSS and their response to changes in RH and temperature. The ability to combine these two techniques in a precisely controlled environment is a significant advancement for investigating coupled water and polymer dynamics, offering a deeper and more holistic understanding of soft-matter systems.

Of course, the presented sample environment is not restricted to soft-matter systems and can be used for any system where hydration and temperature effects are of interest, *e.g.* for superconductivity in oxides, humidity-dependent degradation/phase stability of crystalline materials or ionic conductivity in perovskites (Schaak *et al.*, 2003 ▸; Yun *et al.*, 2018 ▸; Fop *et al.*, 2021 ▸).

## Data Availability

The data that support the findings of this study are available from the corresponding author upon reasonable request.
